# Familial aggregation of anxiety and depression in the community: the role of adolescents’ self-esteem and physical activity level (the HUNT Study)

**DOI:** 10.1186/s12889-015-1431-0

**Published:** 2015-02-04

**Authors:** Ingunn Ranøyen, Frode Stenseng, Christian A Klöckner, Jan Wallander, Thomas Jozefiak

**Affiliations:** Regional Centre for Child and Youth Mental Health and Child Welfare (RKBU), Faculty of Medicine, Norwegian University of Science and Technology, Pb. 8905, Medisinsk teknisk forskningssenter (MTFS), NO-7491, Trondheim, Norway; NTNU Social Research, Trondheim, Norway; Department of Psychology, Norwegian University of Science and Technology, Trondheim, Norway; Psychological Sciences, School of Social Sciences, Humanities and Arts, and Health Sciences Research Institute, University of California, Merced, CA US; Department of Child and Adolescent Psychiatry, St. Olav’s Hospital, Trondheim, Norway

**Keywords:** Intergenerational transmission, Internalising problems, Adolescence, Self-esteem, Exercise, Structural equation modeling

## Abstract

**Background:**

Symptoms of anxiety and depression are significantly associated in parents and children, but few studies have examined associations between recurrent parental problems and offspring symptoms, and fathers have rarely been included in these studies. Additionally, few have investigated factors that may protect against familial aggregation of anxiety and depression. The aims of the present study are to examine the associations between recurrent parental anxiety/depression over a ten-year time span and offspring anxiety/depression in adolescence and to test whether two factors proposed to be inversely related to anxiety and depression, namely, adolescent self-esteem and physical activity, may moderate and mediate the transmission of anxiety/depression.

**Methods:**

This study used data from two waves of a Norwegian community study (the HUNT study) consisting of 5,732 adolescents, ages 13–18, (mean age = 15.8, 50.3% girls) who had one (N = 1,761 mothers; N = 742 fathers) or both parents (N = 3,229) participating in the second wave. In the first wave, 78% of the parents also participated. The adolescents completed self-reported questionnaires on self-esteem, physical activity, and symptoms of anxiety/depression, whereas parents reported on their own anxiety/depressive symptoms. The data were analysed with structural equation modeling.

**Results:**

The presence of parental anxiety/depression when offspring were of a preschool age predicted offspring anxiety/depression when they reached adolescence, but these associations were entirely mediated by current parental symptoms. Self-esteem partly mediated the associations between anxiety/depression in parents and offspring. No sex differences were found. Physical activity moderated the direct associations between anxiety/depression in mothers and offspring, whereas no moderating effect was evident with regard to paternal anxiety/depression.

**Conclusions:**

These findings suggest that children of parents with anxiety/depression problems are at a sustained risk for mental health problems due to the apparent 10-year stability of both maternal and paternal anxiety/depression. Thus, preventing familial aggregation of these problems as early as possible seems vital. The associations between parental and offspring anxiety/depression were partially mediated by offspring self-esteem and were moderated by physical activity. Hence, prevention and treatment efforts could be aimed at increasing self-esteem and encouraging physical activity in vulnerable children of parents with anxiety/depression.

## Background

Mental health problems, such as anxiety and depression, are significantly associated in parents and children [1-4]. These problems “run in the family”, so to speak. The mechanisms of such familial aggregations are most likely very complex and include genetic, environmental and epigenetic processes [5-7]. Most studies have focused on the mechanisms that enforce these processes [8], but few have investigated factors that may protect against such aggregation [1,4]. According to the Roadmap for Mental Health Research in Europe (ROAMER), such factors are important in preventing mental disorders but have too seldom been considered in health sciences [9]. Although associations between positive psychological factors and an adolescent’s mental health have been established (e.g., [10,11]), longitudinal studies that include data on both parents’ and adolescents’ mental health are lacking. Therefore, in the present study, we investigated the relevance of two factors proposed to be inversely related to anxiety and depression [12-14] that may protect against familial aggregation of these problems: self-esteem and physical activity. Specifically, we tested how these factors may moderate and mediate the transmission of mental health problems. We took advantage of the material from a large community study (the HUNT Study), including maternal and paternal mental health data from two measurement points in addition to their offspring’s mental health data at the second measure point.

### Familial aggregation of anxiety and depression

Having a parent with mental health problems, such as anxiety or depression, is one of the most important risk factors for developing such problems [1,8,15-17]. The timing of these problems in both parents and offspring seems to affect the risk for problems in other family members. Children experiencing maternal depression at any time point before the age of 10 have an elevated risk for depression as an adolescent [18]. However, the results from one study indicated that the direction of the relationship between depressive symptoms in mothers and offspring at ages 5–7 may be reciprocal [19], whereas in adolescence, maternal depression most likely leads to offspring depression [20]. Recurrent problems may be indications of the severity and chronicity of a disorder [18,21], and recurrent maternal depression is related to an even higher risk of mental health problems in offspring [21,22], but fathers have rarely been included in such studies. In general, fathers are underrepresented in studies on familial aggregation of anxiety and depression [1,23]. This is a problem because fathers obviously contribute 50% of their offspring’s genes and are increasingly more involved in child care [4]. Furthermore, few studies have taken into account the fact that anxiety and depression are highly comorbid. Thus, we lack information on whether current symptoms in both parents mediate the relationship between a parental history of anxiety and depression and offspring anxiety and depression [22].

### Self-esteem among adolescents

Global self-esteem refers to a person’s evaluation of overall self-worth and self-knowledge [12,24]. Empirical research has indicated that a low global self-esteem predicts depression, while depression does not predict low self-esteem [25-28]. For anxiety, the findings are mixed, although the associations appear to be more bidirectional [25,28]. Several studies have shown that the children of depressed parents have a lower self-esteem than the children of non-depressed parents [2,29-31]. Another study showed that depressed mothers expressing negative affectivity about their children have children with a lower global self-worth and more psychopathology than the children of non-depressed mothers [32]. In contrast, a higher self-esteem in the offspring of depressed parents is found to be the most important predictor of both the absence of a psychiatric diagnosis and high global functioning 2, 10, and 20 years later [33].

The role of self-esteem in a family perspective can be explained by an encompassing developmental model detailing the mechanisms involved in the intergenerational transmission of depression [34,35]. According to this theory, the children of depressed mothers may inherit genetic predispositions towards depression and be born with dysfunctional neuroregulations. In addition, these children may be exposed to a stressful family environment and the negative cognitions, behaviours, and/or affects of the depressed parent. These factors are assumed to create certain vulnerabilities in children - including low self-esteem - which can contribute to depression. Whereas studies have examined a straightforward relationship between parental depression and child self-esteem, to our knowledge, only two studies have examined self-esteem as a mediator in relation to depression in a family perspective. One study showed that psychological control in depressed mothers was associated with depressive symptoms in offspring, and these relations were partially mediated by offspring self-esteem [36]. Another study found that maternal depression partly predicted offspring self-esteem, which in turn predicted offspring depression [22]. However, these studies were limited by only including mothers. Thus, we lack studies examining whether self-esteem mediates the associations between maternal, paternal and offspring anxiety/depression.

### Physical activity level and mental health

Physical activity, here defined as aerobic activity of at least moderate intensity resulting in noticeably increased heart rate or rapid breathing [37], is assumed to protect against anxiety, depression and low self-esteem [38,39]. For example, physical activity may be protective by enhancing an individual’s autonomy, competence, and social interaction, as proposed by the self-determination theory [40,41]. This is intriguing because compared to many other protective factors, most individuals can engage in physical activity, and motivation for physical activity can be altered with public health interventions [40]. Empirical findings on the benefits of exercise on anxiety and depression are, however, inconsistent. Some studies have found a small effect of exercise on anxiety and depression among adolescents [42-45], but the clinical significance of these associations is questionable due to numerous methodological limitations [14,46]. There are also a few studies showing that physical activity increases self-esteem in adolescents [47-49]. However, systematic reviews have concluded that the evidence base is currently too scarce to indicate that physical activity affects either self-esteem or anxiety/depression among adolescents [46,47]. Furthermore, the psychosocial mechanisms explaining the links between physical activity and anxiety/depression need to be examined [50]. To our knowledge, this is the first study to examine whether adolescents’ physical activity moderates the associations between parental and adolescent anxiety/depression and self-esteem.

### Aims and research questions

**Figure 1:**
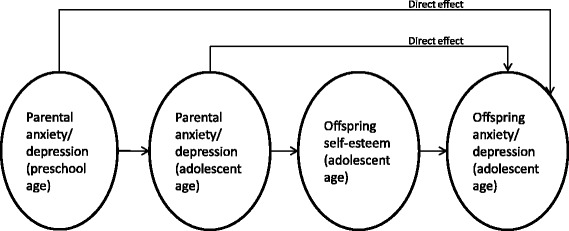
**Conceptual model examined in the present study.** We tested for invariance across sexes and different levels of offspring physical activity.

Based on the reviewed literature, the aims of the present study were to examine the associations between recurrent parental anxiety/depression over a ten-year time span and offspring anxiety/depression in adolescence and to explore the role of adolescent self-esteem and physical activity in familial aggregation of anxiety/depression in a large community sample. The following research questions were addressed: (1) Are maternal and paternal anxiety/depression when offspring are of a preschool age associated with offspring anxiety/depression when they reach adolescence; (2) are the associations between maternal and paternal anxiety/depression when offspring are of a preschool age and offspring’s anxiety/depression in adolescence mediated by (current) maternal and paternal symptoms in adolescence; (3) are the associations between parental and adolescent anxiety/depression mediated by offspring self-esteem; and (4) are the associations between parental and adolescent anxiety/depression and self-esteem moderated by offspring physical activity levels? We will also explore sex differences in these relationships, and the model depicted in Figure 1 will guide our research.

## Methods

### Design and procedures

Our study is based on parental data from the questionnaire portion of the second and third Nord-Trøndelag Health Study (termed HUNT2 and HUNT3, respectively) and offspring data from the questionnaire portion of HUNT3 only. The HUNT study is a large, total population health survey comprised of questionnaires and clinical examinations conducted in the county of Nord-Trøndelag, Norway. HUNT2 was conducted from 1995–1997, and HUNT3 was conducted from 2006–2008. In both waves, all the inhabitants in the county above the age of 12 were invited to participate in the study without any exclusion criteria. There are 131,000 inhabitants of primarily Norwegian descent in the county. In both HUNT2 and HUNT3, the adult participants aged 20 and older received a letter, information brochure, and questionnaire by mail. They completed the questionnaire at home and delivered it at the time of the subsequent clinical examination. More details on the adult sample are published elsewhere [51].

For adolescent participants ages 13–18, the questionnaire was administered during a class in all junior high and high schools in the county. Teachers were asked to read the questions aloud to adolescents with problems reading or answering the questionnaire. The adolescents who were temporarily away from school on the day of survey completion received the survey approximately one month later during a health exam that was also part of HUNT3. Adolescents not attending school at all (4.2%) received the survey by mail. A detailed description of the adolescent sample is published elsewhere [52]. Data from the adolescents were linked to data from their biological or adoptive parents using each citizen’s unique personal national ID number.

### Participants

HUNT3 included 8200 adolescents in junior high and high schools (78% response rate). Most of the non-responders were not in school and were older, more often boys, and if they were in school, they attended vocational rather than academic classes [52]. The participants aged 12 (n = 27), 19 (n = 219), and 20 (n = 41) were excluded from further analyses due to low age sample sizes. HUNT3 included 50,827 adult participants ages 19 through 101.

**Figure 2:**
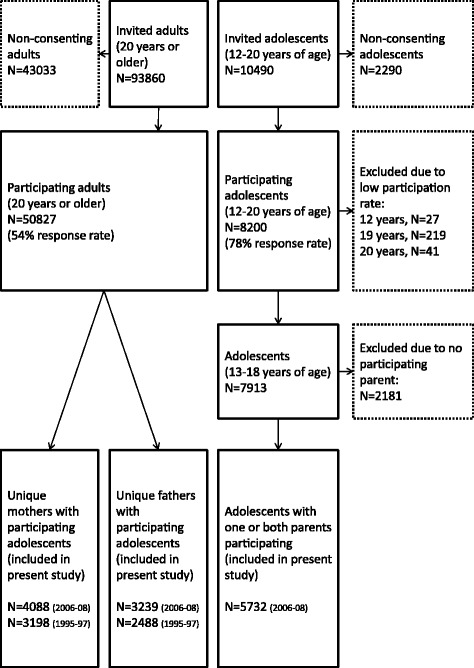
**Flowchart of the participants in the present study.**

The sample for the present study was formed by identifying those adolescents having at least one biological or adoptive parent participating in the study, which excluded 2181 (28%) adolescents. Thus, our analysis sample consisted of 5732 adolescents, of whom 3229 had both parents, 1761 had only their mother, and 742 had only their father participating. Twenty-seven of these adolescents were adopted. 3198 of the mothers (78%) and 2488 of the fathers (77%) participated in HUNT2. The participant flow chart is shown in Figure 2.

### Measures

#### Adolescent variables

*Anxiety*/*depression symptoms* were measured using the Symptom Check List-5 (SCL-5), which consists of five items from the 25-item version [53]. Whereas the SCL-5 has shown very high correlations (*r* = .92) with the SCL-25 and a satisfactory reliability [54,55], a distinction between anxiety and depressive symptoms is not possible from this reduced item set [56]. However, the odds ratio for comorbidity between these problems is estimated to be 28 [57]. The tripartite model of anxiety and depression, which has received much empirical support, posits that anxiety and depression often occur together due to shared genetic factors and a common distress factor marked by negative affect [58]. This is evident in that some symptoms are common to both anxiety and depression, whereas other symptoms are unique to either anxiety or depression. Each symptom was rated on a 4-point scale (1 = *not bothered*, 4 = *very bothered*). The composite reliability (CR) was .886 and .879 for daughters and sons, respectively.

*Self*-*esteem* was measured with a four-item version of the Rosenberg Self-Esteem Scale [24]. The items correlate highly with the original scale (0.95) [59], which has demonstrated construct validity as a measure of self-esteem in a large body of literature. The items were rated on a 4-point scale (1 = *totally agree*, 4 = *totally disagree*). The CR was .851 and .792 for daughters and sons, respectively.

*Physical activity* was measured with one question that was also used in the World Health Organization Health Behaviour in School-aged Children (WHO HBSC) survey [60]: “Apart from the average school day, how many days a week do you play sports or exercise to the point where you breathe heavily and/or sweat?” The question had eight response alternatives: “every day”, “4–6 days a week”, “2–3 days a week”, “one day a week”, “not every week, but at least once every 14th day”, “not every 14th day, but at least once a month”, “less than once a month” and “never”. The question has shown acceptable validity demonstrated by correlations with physical fitness measured by maximal oxygen uptake (VO_2_ peak), which is often considered the gold standard in assessing physical fitness [61]. In Norway, adolescents in this age range also have at least 2–3 school lessons per week in Physical Education. Research suggests that differences in adherence, motivation, enjoyment, and genetics exist between individuals who choose to exercise regularly and those who do not [14,62,63]. Thus, we dichotomised the answers into regular and low physical activity based on regular exercise outside of school hours: “regular activity” represented “one day a week” or more, whereas “low activity” represented “less than once a week”. Such dichotomisation has been used in other studies to detect differences between low and regularly active groups (e.g., [64]).

#### Parental variables

*Symptoms of anxiety*/*depression* were measured by the Cohort Norway Mental Health Index (CONOR-MHI) [65], which consists of seven items based on the General Health Questionnaire [66] and the Hopkins Symptom Check List [53]. The CONOR-MHI correlates highly with both the Symptom Check List-10 (SCL-10) (*r* = .82) and the Hospital Anxiety and Depression Scale (HADS) [67] (*r* = .91), but separating anxiety and depressive symptoms is not possible [65]. Examples of items include “Have you, in the course of the last two weeks, felt nervous and unsettled?” and “…happy and optimistic?” Each item was rated on a 4-point scale (1 = *no*, 4 = *very much*). To be able to describe a group with high anxiety/depression, we used the mean cut-off value of ≥ 2.15, which was established from cut-off values of the SCL-10 and HADS that were shown to identify anxiety disorders and major depressive disorder [65]. For daughters and sons, respectively, the CR for maternal anxiety/depression when offspring were adolescents was .932 and .915, while at preschool age the CR was .920 and 908; for paternal anxiety/depression, the CR when offspring were adolescents was .923 and .914, and at preschool age the CR was .898 and .909.

For descriptive purposes, *Physical activity* in parents was measured by one question: “How often do you exercise?” The question had five response alternatives: “never”, “less than once a week”, “once a week”, “2–3 times a week”, and “approximately every day”.

### Ethics

All participants in the study, and at least one parent when the adolescent was under 16 years of age, signed a written informed consent to participate. This study was approved by the Regional Committee for Medical and Health Research Ethics (reference number 4.2007.2416).

### Statistics

To reduce the likelihood of respondent fatigue, short forms of established measurement scales were used in this survey. To ensure the validity of the short forms, we analysed the data by structural equation modeling, as recommended [68]. The models were estimated with the weighted least squares mean and variance adjusted estimator (WLSMV) due to the categorical and non-normal nature of the indicators. To explore sex differences, we estimated multi-group models separating daughters and sons and tested possible differences with Wald tests of parameter constraints.

Prior to examining the aims of the study by estimating the path models, measurement invariance was explored. By adding increasingly more restrictions, configural, metric, and scalar measurement invariance were tested [69]. When scalar invariance is established, measurement invariance is assumed [70]. This indicates that the sex differences in the means of the observed items stem from differences in the means of the latent variables. Within the latent variable modeling framework, several fit indices are usually examined. A non-significant *χ*^2^- statistic, *CFI*- and *TLI*-values > .95, and *RMSEA*-values < .06 indicate a good model fit [71]. When evaluating measurement invariance, we also examined *CFI*-differences and *RMSEA*-differences, as has been recommended [72]^a^, because *χ*^2^-difference tests are sensitive to sample size and model complexity [73]. *ΔCFI* ≥ .010 and *ΔRMSEA* ≥ .015 indicate measurement noninvariance [72]. We used composite reliability (CR) to evaluate internal consistency because unlike the coefficient alpha, CR does not assume that all items are equally good indicators of the latent variable measured [74]. An estimated *CR* ≥ .7 indicates a good reliability, and a *CR* of .6 - .7 is considered acceptable.

Four structural models were tested. When estimating indirect paths in the mediation models, we followed the recommended procedures with 1,000 bootstrap samples [75]. Multi-group mediation models were used to test moderated mediation because such analyses with latent variables often result in model non-convergence. As recommended [75], the indirect effects, the difference between the direct and indirect effects and between the effects across groups were tested by computing new parameters and examining their statistical significance. Portions of our sample were hierarchically structured. A total of 2062 adolescents had at least one sibling also participating, whereas 3670 adolescents did not have a participating sibling. Standard errors and *χ*^2^-tests were corrected for this potential cluster effect in the analyses when possible^b^. Adolescent age was associated with symptoms of anxiety/depression but not with self-esteem. Thus, we adjusted for age in the paths including adolescent anxiety/depression. Parental marital status was not related to our dependent variables, and we did not adjust for this variable.

The sample size was large; however, the magnitude of effects was small because most participants in community samples were healthy. Thus, we considered two-sided p-values < .05 as statistically significant. We used IBM SPSS Statistics 19 for the descriptive statistics. The main analyses were performed using Mplus, version 7.11 [76]. There was a low rate of missing values in the data set (≤4%). Thus, all missing values were handled by the full information maximum likelihood procedure (FIML) in Mplus.

## Results

### Demographic and sample characteristics

Characteristics of the sample have been reported in a previous publication [2]. The proportions of adolescent daughters (50.3%) and sons (49.7%) were approximately the same. There was no difference in the mean age for daughters (M = 15.8; *SD* = 1.7) and sons (M = 15.8; *SD* = 1.6). One-third of the adolescents reported having parents not living together. There were more mothers than fathers participating (56% vs. 44%). As expected, the mothers were significantly younger (M = 44.2; *SD* = 5.2) than the fathers (M = 47.6; *SD* = 5.9) (*t* (7306) = 26.104, *p* < .001). Consistent with the overall Norwegian population, the mothers had higher levels of education (M = 4.43; *SD* = 1.54) than the fathers (M = 4.21; *SD* = 1.47) (*t* (7322) = 6.188, *p* < .001). The most frequent educational level was upper secondary education, which was somewhat higher than in the general Norwegian population [77]. When the offspring were of a preschool age, 7.5% of the mothers and 4.0% of the fathers were identified with anxiety/depression by scoring above the cut-off value, whereas when the offspring were adolescents, the corresponding proportions were 6.8% for the mothers and 5.9% for the fathers. 14.5% (N = 591) of mothers and 27.4% (N = 889) of fathers reported exercising less than once a week (low physical activity), whereas 84.8% (N = 3466) of mothers and 71.3% (N = 2310) of fathers reported exercising once a week or more (regular physical activity). Parental anxiety/depression was negatively associated with parental physical activity for both the mothers (β (*SE)* = −.108 (.018), *p* < .001) and the fathers (β *(SE)* = −.071 (.021), *p* = .001). A total of 87.6% of the adolescents were in the regular physical activity group (N = 5023), whereas 12.4% were in the low physical activity group (N = 709). There were no sex differences in the regular (50.2% daughters) and low (50.9% daughters) activity groups. In a previous study, we found that adolescents who were excluded due to not having parents participating in the study had significantly more symptoms of anxiety/depression and a lower self-esteem than those with participating parents, but the effect sizes for these differences were very small (Cohen’s *d* < .14) [2]. Adolescents without participating parents were also significantly less physically active than adolescents with participating parents (*t* (7787) = 3.319, *p* < .01), but the effect size for this difference was also very small (*d* = .08; *r* = .04).

### Measurement models

Most fit indices for the measurement models indicated a good fit of all the models, but the *χ*^2^-statistic was significant, as expected for the large sample size and the complex model ([*configural* model: *χ*^2^ (1228, N = 5732) = 6333, *p* <. 001; *CFI* = .951; *TLI* = .947; *RMSEA* = .038]; [*metric* model: *χ*^2^ (1259, N = 5732) = 6435, *p* <. 001; *CFI* = .951; *TLI* = .948; *RMSEA* = .038]; [*scalar* model: *χ*^2^ (1326, N = 5732) = 6450, *p* <. 001; *CFI* = .951; *TLI* = .951; *RMSEA* = .037]). Neither *ΔCFI* nor *ΔRMSEA* indicated a worse fit to the data when more restrictions were added (*ΔCFI* = .000; *ΔRMSEA = .001*). Thus, scalar measurement invariance was established. Also indicating a good model fit, the unstandardised factor loadings in the scalar model were satisfactory and statistically significant (*p* < .001) for all indicators: self-esteem (.890 ≤ *b* ≤ 1.000); offspring anxiety/depression (.985 ≤ *b* ≤ 1.083); maternal anxiety/depression when offspring were adolescents (.632 ≤ *b* ≤ 1.000); paternal anxiety/depression when offspring were adolescents (.715 ≤ *b* ≤ 1.000); maternal anxiety/depression when offspring were of a preschool age (.720 ≤ *b* ≤ 1.000); paternal anxiety/depression when offspring were of a preschool age (.705 ≤ *b* ≤ 1.000). Further details of the measurement models are available from the authors. Table 1 displays the correlations among the variables.

**Table 1:** **Correlations of the variables (standard errors in parentheses)**

**Daughters/sons**	**1**	**2**	**3**	**4**	**5**	**6**	**7**
1. Offspring anxiety/depression	-	.571 (.020)	.119 (.024)	-.118 (.028)	-.138 (.031)	-.103 (.031)	-.093 (.035)
2. Offspring self-esteem	.640 (.015)	-	.225 (.022)	-.075 (.028)	-.107 (.030)	-.066 (.031)	-.092 (.033)
3. Offspring physical activity	.134 (.022)	.195 (.021)	-	-.071 (.024)	-.076 (.026)	-.069 (.027)	-.080 (.030)
4. Maternal anxiety/depression	-.129 (.026)	-.106 (.025)	-.027 (.023)^n.s.^	-	.231 (.035)	.506 (.025)	.149 (.038)
5. Paternal anxiety/depression	-.202 (.027)	-.102 (.029)	-.131 (.026)	.263 (.032)	-	.209 (.038)	.551 (.030)
6. Maternal preschool anxiety/depression	-.090 (.027)	-.109 (.029)	-.055 (.026)	.495 (.025)	.146 (.036)	-	.323 (.040)
7. Paternal preschool anxiety/depression	-.096 (.033)	-.138 (.034)	-.107 (.031)	.060 (.037)^n.s.^	.546 (.027)	.208 (.039)	-

Daughters are below the diagonal and sons are above the diagonal. All correlations were statistically significant (*p* < .05), except if otherwise indicated by a superscript.

### Is parental anxiety/depression when offspring were of a preschool age associated with offspring anxiety/depression in adolescence?

**Figure 3:**
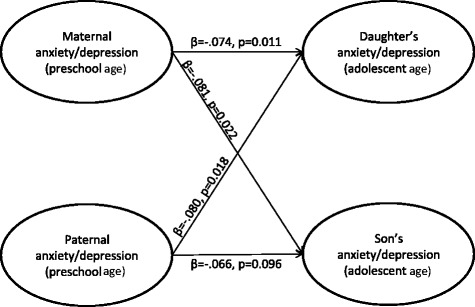
**Associations between parental anxiety/depression when offspring were at preschool age and offspring anxiety/depression in adolescence.** Standardised coefficients and p values are presented. Associations for daughters are presented in the upper part of the figure; associations for sons are presented in the lower part of the figure.

Both maternal and paternal symptoms of anxiety/depression when offspring were of a preschool age were weakly but significantly associated with such symptoms in adolescent offspring approximately ten years later (see Figure 3). Except for a significant *χ*^2^-test, which was expected due to the large sample size, the fit indices for the model were good (*χ*^2^ (348, N = 5707) = 1787, *p* < .001; *CFI* = .968; *TLI* = .969; *RMSEA* = .038). Although the path from paternal symptoms to sons’ symptoms was not significant, this path was not significantly different for daughters and sons (Wald test of parameter constraints (1) = 0.159, *p* = .69). When constraining the parameters to be equal for daughters and sons, the model fit was even better, and all the paths were significant. This indicates that both maternal and paternal anxiety/depression when offspring were of a preschool age were associated with such symptoms in both daughters and sons ten years later.

### Are the associations between parental anxiety/depression when offspring were of a preschool age and offspring anxiety/depression in adolescence mediated by current parental symptoms?

**Figure 4:**
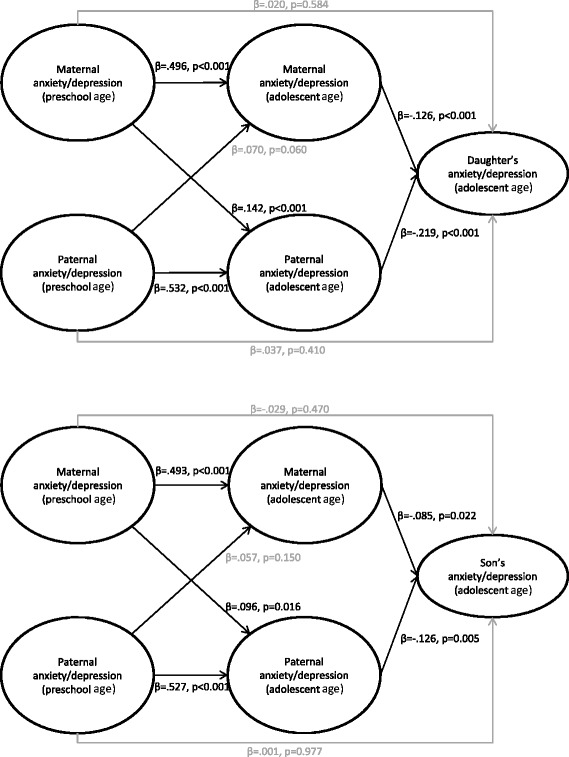
**Parental anxiety/depression in adolescence as a mediator.** The associations between parental anxiety/depression when offspring were preschool aged and offspring anxiety/depression in adolescence mediated by parental anxiety/depression in adolescence for daughters (the upper part of the figure) and sons (the lower part of the figure), presented with standardised coefficients and p values (statistically significant paths are in black (p < .05), non-significant paths are in grey).

When including current parental anxiety/depression, the associations between an adolescent offspring’s symptoms of anxiety and depression and such symptoms in parents when offspring were of a preschool age were fully mediated by parental anxiety/depression in adolescence (see Figure 4). Additionally, the fit indices were good for this model (*χ*^2^ (1124, N = 5732) = 5819, *p* < .001; *CFI* = .950; *TLI* = .951; *RMSEA* = .038). There were no significant sex differences, indicating that the associations between both maternal and paternal anxiety/depression when offspring were of a preschool age and daughters’ and sons’ anxiety/depression in adolescence were mediated by current maternal and paternal anxiety/depression.

### Are familial associations of anxiety/depression mediated by adolescent self-esteem?

**Figure 5:**
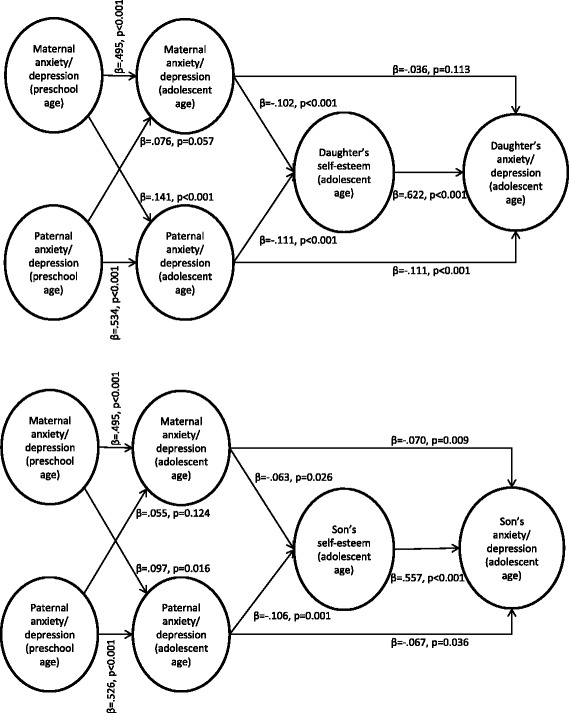
**Adolescent self-esteem as a mediator.** The associations between parental anxiety/depression and adolescent offspring anxiety/depression mediated by adolescent self-esteem presented with standardised coefficients and p values. Associations for daughters are presented in the upper part of the figure; associations for sons are presented in the lower part of the figure.

An initial model showed that parental anxiety/depression when offspring was of a preschool age was not associated with later adolescent self-esteem for either sex. Thus, we analysed a new model excluding these paths and only including current parental anxiety/depression, shown in Figure 5. The fit indices for this model were good (*χ*^2^ (1408, N = 5732) = 6397, *p* < .001; *CFI* = .953; *TLI* = .953; *RMSEA* = .035). The results from this model (see Figure 5) showed that adolescent self-esteem was significantly associated with adolescent anxiety/depression for both sexes. For both daughters and sons, the indirect paths from both maternal (daughters: *β (SE)* = −.064 (.016), *p* < .001; sons: *β (SE)* = −.035 (.016), *p* = .028) and paternal anxiety/depression (daughters: *β (SE)* = −.069 (.018), *p* < .001; sons: *β (SE)* = −.059 (.016), *p* = .001) to offspring anxiety/depression via offspring self-esteem were also significant. For sons, the direct paths from both maternal and parental anxiety/depression to sons’ anxiety/depression were still significant. For daughters, the direct path from paternal anxiety/depression to daughters’ anxiety/depression was still significant, whereas the direct path from maternal anxiety/depression was not. This path was, however, not significantly different for daughters and sons (Wald test of parameter constraints (1) = 1.181, *p* = .28), and when constraining the parameters to be equal for both daughters and sons, both maternal and paternal anxiety/depression were significantly associated with anxiety/depression in both daughters and sons. Thus, parental anxiety/depression was still significantly directly associated with offspring anxiety/depression in adolescence. The direct and indirect effects were not significantly different either for daughters (*b (SE)* = .021 (.026), *p* = .412) or sons (*b (SE)* = −.028 (.031), *p* = .368). Thus, parental and adolescent anxiety/depression were partly directly associated and partly mediated by a low adolescent self-esteem.

### Does adolescent physical activity protect against familial aggregation of anxiety/depression?

Finally, we examined whether adolescent physical activity moderated the direct or indirect paths associating parental anxiety/depression with offspring anxiety/depression. We conducted multi-group analyses with two groups consisting of adolescents who responded “low” vs. “regular” on self-initiated physical activity. In this model, we did not distinguish between daughters and sons because there were no significant sex differences in the previous model (reported above) and because the proportion of daughters and sons were the same in both the low (50.9% daughters) and the regular (50.2% daughters) physical activity group (*Z* = −0.353; *p* = .726). The fit indices for the model were good (*χ*^2^ (553, N = 5732) = 3973, *p* < .001; *CFI* = .955; *TLI* = .955; *RMSEA* = .046).

**Figure 6:**
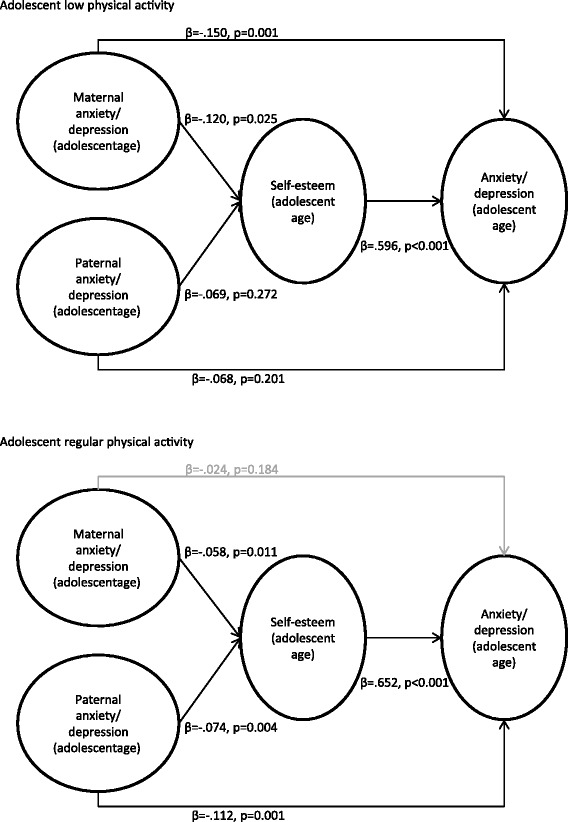
**Adolescent physical activity level as a moderator.** The associations between parental anxiety/depression and offspring anxiety/depression in adolescence mediated by adolescent self-esteem for the two physical activity groups (low and regular) are presented with standardised coefficients and p values (statistically significant paths are in black (p < .05), non-significant paths are in grey). Associations for the low physical activity group are presented in the upper part of the figure; associations for the regular physical activity group are presented in the lower part of the figure.

This model (see Figure 6) showed that the difference between the direct associations between maternal and offspring anxiety/depression was significant in the low vs. regular activity groups (*b (SE)* = −.124 (.051), *p* = .015). This indicates that maternal and offspring anxiety/depression were not directly associated among adolescents exercising at least once a week outside of school hours. The drop in estimates between maternal anxiety/depression and offspring self-esteem was, however, not significant (*b (SE)* = −.057 (.054), *p* = .291). The direct and indirect paths were significantly different (*b (SE)* = .628 (.039), *p* < .001), indicating that regular physical activity moderated the direct path between maternal and offspring anxiety/depression but not the indirect paths between maternal and offspring anxiety/depression via offspring self-esteem. Physical activity did not moderate the associations between paternal and adolescent anxiety/depression either directly (*b (SE)* = −.007 (.058), *p* = .911) or indirectly via offspring self-esteem (*b (SE)* = −.014 (.039), *p* = .710). Thus, only the direct path between maternal and adolescent anxiety/depression was moderated by physical activity. Although the indirect paths between parental and offspring anxiety/depression via self-esteem were not moderated, the regular activity group did have a significantly higher self-esteem than the low activity group (*b (SE)* = .346 (.043), *p* < .001).

## Discussion

Our results showed that parental anxiety/depression when offspring were of a preschool age is associated with such problems in adolescent offspring, but these associations are entirely mediated by current parental symptoms. The familial associations are evident for both daughters and sons regardless of whether anxiety/depression is present in mothers or fathers, thus extending previous research findings to also include paternal symptoms. Our findings also indicated that adolescent self-esteem partly mediates the associations between anxiety/depression in parents and their offspring. Finally, physical activity seems to moderate the direct associations between anxiety/depression in mothers and offspring, whereas no such moderating effect is evident with regard to paternal anxiety/depression. To our knowledge, the present study was the first to suggest that physical activity may protect against familial aggregation of anxiety/depression.

### Familial aggregation of anxiety/depression

Parental anxiety/depression when offspring were of a preschool age was weakly associated with such problems in offspring in adolescence, and this relationship was entirely mediated by current anxiety/depression in parents. This may imply that the stability of parental mental health problems leads to problems in offspring, although our findings may also support previous research suggesting a reciprocal relationship between at least maternal and offspring depressive symptoms in children ages 5–7 [19]. Whereas we lacked information about offspring mental health at a preschool age to test this more directly, this directionality is partly supported by studies showing that improvement in parental depression leads to improvement in offspring psychopathology [78,79], whereas continued maternal symptoms are related to fewer declines or elevations of symptoms in offspring [20]. That this effect was fully mediated by parental anxiety/depression in adolescence in our study suggests a 10-year stability of these problems among adults. Furthermore, our findings may reflect the chronic nature of anxiety and depression, which has also been reported in previous studies [80,81]. Studies show that recurrent maternal depression is related to a higher risk of such problems in offspring [22], and nearly 60% of adolescents with anxiety and depression have further episodes in adulthood [82]. These findings point to the risk associated with stability, and likely chronicity, in parental anxiety/depression, and indicates that it is vital to prevent familial aggregation of these problems as early as possible. Our results may suggest that parental psychopathology in the early years of life may have long-term implications for offspring and trigger negative developmental cascades. Thus, health professionals working with adults with anxiety/depression should be aware of the possible negative consequences for offspring and seek to prevent the development of such symptoms in the offspring. Furthermore, these findings emphasize the importance of addressing mental health problems in a familial context. Our study expands on previous research by examining associations between recurrent paternal anxiety/depression and such problems in offspring. We found that both maternal and paternal problems were associated with offspring anxiety/depression, and there were no significant sex differences. This underscores the importance of including both mothers and fathers when studying familial aggregation of mental health problems and highlights the fact that paternal anxiety and depression is just as important for the mental well-being of adolescents as maternal problems. Thus, our findings point to the importance of assessing the mental status of the entire immediate family both in clinical practice and in research, as we also reported in a previous study [2].

### Self-esteem as a mediator in familial aggregation of anxiety/depression

The fact that low self-esteem partially mediated the associations between parental and offspring anxiety/depression is in accordance with the developmental model detailing that children of depressed mothers are exposed to risks that may eventually create a vulnerability for low self-esteem, which over time increases the risk for depression, especially in adolescence [34,35]. Our findings also support previous studies showing that children of parents with depression have lower self-esteem than children of healthy parents [2,29-32]. Likewise, our results support previous findings that offspring self-worth partly mediates the associations between maternal and offspring depression [22]. Again, our study demonstrates the role of fathers by indicating that self-esteem also partly mediates associations between paternal and offspring symptoms of anxiety/depression in addition to associations between maternal and offspring symptoms. These results also emphasise the importance of including both mothers and fathers in studies of familial aggregation of mental health problems. Additionally, our findings indicated that self-esteem partly mediates the associations between symptoms of both anxiety and depression, as suggested in the tripartite model [58], and not only symptoms of depression. Future research should examine whether self-esteem also mediates parental and offspring anxiety. Because different anxiety disorders are differently related to the factors of the tripartite model [83,84], it will also be important to distinguish among anxiety disorders when further examining the etiological role of self-esteem.

### Physical activity as a moderator in familial aggregation of anxiety/depression

Our findings suggested that physical activity moderates the direct associations between maternal and offspring anxiety/depression but not between paternal and offspring anxiety/depression. Additionally, physical activity did not moderate the indirect paths associating parental and offspring anxiety/depression via self-esteem. As we have not been able to find studies examining the role of physical activity in familial aggregation of anxiety and depression, these findings should serve as the basis for further research on this subject. Nonetheless, our findings support studies showing an inverse association between physical activity and anxiety/depression [42,43,45] because maternal and adolescent anxiety/depression were not associated in adolescents reporting self-initiated physical activity beyond that prescribed in school. This indicates that physical activity can act as an important protective factor against anxiety/depression in adolescents with mothers with such problems. Hence, beyond treating adults with symptoms of anxiety/depression, it is important that professionals working in the primary care services also inform about the positive effects of and encourage physical activity in the family.

Physical activity did not moderate the associations between paternal and offspring anxiety/depression. This may be explained by the fact that a father’s physical activity is shown to be one of the strongest predictors for physical activity in adolescents, whereas a mother’s physical activity appears less important [85-87]. This is corroborated by the finding that fathers with anxiety/depression were significantly less physically active than healthy fathers. Thus, the offspring of fathers with anxiety/depression may also be less physically active, whereas in families with a healthy and physically active father, adolescents most likely exercise more. Further research is necessary to examine these hypotheses.

Biochemical, physiological and psychological mechanisms explaining the relationships between physical activity and anxiety/depression have been proposed [14,88,89]. One of the most important psychological mechanisms is the distraction hypothesis, which posits that the “time out” from difficult thoughts during exercise results in reduced anxiety and depression [90]. Another possible mechanism stems from the self-determination theory [41], which claims that physical activity may increase feelings of autonomy, competence and provide more possibilities for social interaction, which are properties shown to increase self-esteem and reduce anxiety and depression [91]. Finally, physical activity may reduce anxiety and depression by increasing self-esteem [49], but in this study, physical activity did not affect the paths associating parental and offspring anxiety/depression via self-esteem. However, the regular activity group did have a significantly higher self-esteem than the low activity group. Physical activity may potentially increase self-esteem or vice versa, but further research is necessary to explore the precise nature of these relationships.

As mentioned previously, there is ambiguity with regard to the effect of physical activity on anxiety and depression in existing research [46]. This study expands on previous research by suggesting that adolescents exercising with moderate to vigorous intensity at least once a week outside of school may be protected against symptoms of anxiety and depression when having a mother with such symptoms. The differential susceptibility hypothesis [92] claiming that children at risk (e.g. due to having parents with anxiety/depression) may be more strongly influenced by positive environmental experiences might be relevant for our findings. Physical activity may be a positive experience affecting children of parents with anxiety/depression more strongly than children of healthy parents, and thus partly account for the previous ambiguous findings in this research area. Future research should examine the possible thresholds for positive consequences of physical activity and the cause-effect relationships between physical activity and symptoms of anxiety/depression further, for example by employing latent class analysis, cross-lagged autoregressive analysis, or possibly conducting a randomized controlled trial.

### Strengths and limitations

The inclusion of a large number of fathers (>3000) is an important addition to previous research examining familial aggregation of anxiety/depression and the mediational role of self-esteem. Additionally, our study is the first that we have been able to find that examines whether physical activity moderates the associations between parental and offspring anxiety/depression. Such investigations examining the factors important for positive mental health have been suggested by the ROAMER project [9]. In addition, we assessed moderated mediation by examining whether physical activity affected the mediational paths from parental to offspring anxiety/depression via offspring self-esteem using advanced statistical methods. Using data from a large community sample with a high response rate also made it possible to examine sex differences without jeopardising the statistical power. There was a low degree of missing values in the present study (<4.0%), which removed the need to use multiple imputation to handle the missing values. Finally, we were able to examine some of the hypotheses using a long-term longitudinal design.

There are several limitations in the current study. First, because this is a correlational study, causal processes cannot be determined. Thus, even though we emphasised the interpretation that parental mental health problems likely lead to problems in offspring, the reverse or reciprocity cannot be ruled out. Additionally, our assumption that self-esteem leads to depression was based on previous research findings and was not examined in the present study. Further longitudinal studies can advance inferences about the direction of associations between the variables measured in our study. Using data from a large community sample with a high response rate would usually indicate representative results, but the adolescents without participating parents in the HUNT3 study had slightly more symptoms of anxiety/depression, had lower self-esteem, and were less physically active than the adolescents with participating parents. This might result in an underestimation of the observed associations in the present study, but the small effect sizes should partially abate that concern. Additionally, the participation rate of adolescents not in school was too low to be representative, possibly leading to an underestimation of associations because this group may have a worse mental health and life style. Also, the generalizability of our findings to other cultures may be limited. Norway is a wealthy country with significant resources available to the whole population, including prominently universal public health insurance coverage and equal status of the sexes. The socioeconomic health inequalities in the county of Nord-Trøndelag are, however, comparable to other countries in Northern Europe [93], suggesting that the findings at least may be representative for other North European countries. Future research should examine whether our findings also can be generalized to other cultures. Although self-reports may be biased, studies generally find self-reports of mental health and physical activity to be valid [94]. With the measures used here, we were not able to differentiate between anxiety and depression. However, because the comorbidity between these problems is high [57], this limitation may be mitigated. Other relevant mediators and moderators were not examined; thus, future research should include more variables to broaden the understanding of familial aggregation of anxiety and depression.

## Conclusions

Our findings suggest that the children of parents with anxiety/depression problems are at a sustained risk for mental health problems due to the chronic nature of these symptoms, as shown by the apparent 10-year stability of both maternal and paternal anxiety/depression. Thus, preventing familial aggregation of these problems as early as possible seems vital. Because our study indicates that the associations between parental and offspring anxiety/depression are partially mediated by offspring self-esteem, efforts could be aimed at increasing self-esteem in vulnerable children as early as possible in life. Engaging in activities that fulfil an individual’s basic needs of autonomy, competence, and relatedness has been shown to increase self-esteem [91]; thus, encouraging such activities may also serve to mitigate familial aggregation of anxiety/depression. Furthermore, because this study is one of the first to suggest that physical activity can reduce familial aggregation of anxiety/depression, this could be an important focus for future prevention and treatment efforts. Thus, clinicians should view symptoms of anxiety and depression as a familial problem, and inform about the possible positive consequences of physical activity and work to increase motivation and possibly prescribe exercise programs for adolescents exercising less than once a week outside of school. From a public health perspective, it seems important to encourage physical activity in the offspring of parents with anxiety/depression, possibly from early in life.

## Endnotes

^a^It should be noted that these recommendations are based on simulation studies using maximum likelihood estimations of continuous data. No standards for such evaluations of alternative fit indices exist for WLSMV estimations [95], although there are some indications that the WLSMV ΔRMSEA performs particularly well [96].

^b^The adjustment for cluster effects is not available with bootstrapping. Thus, we examined the differences between bootstrapped estimates without clustering and non-bootstrapped estimates with clustering. Not adjusting for clustering resulted in lower chi-square values but no evident differences in the standard errors.

## Abbreviations

HUNT: The Nord-Trøndelag Health Study
SCL: Symptom Check List
WHO HBSC: World Health Organization Health Behaviour in School-aged Children
VO_2_ peak: Maximal oxygen uptake
CONOR-MHI: Cohort Norway Mental Health Index
HADS: Hospital Anxiety and Depression Scale
WLSMV: Weighted least squares mean and variance adjusted estimator
CFI: Comparative fit index
TLI: Tucker-Lewis Index
RMSEA: Root mean square error of approximation
CR: Composite reliability
FIML: Full information maximum likelihood
